# The Cyclopædia of Practical Medicine

**Published:** 1844-08

**Authors:** 


ART. VIII.—*The Cyclopædia of Practical Medicine.* Edited by JOHN FORBES, M.D., F.R.S., ALEX. TWEEDIE, M.D., F.R.S., and JOHN CONOLLY, M.D. Revised, with additions, by ROBLEY DUNGLISON, M.D. Parts V., VI., and VII. Philadelphia: Lea & Blanchard. 1844.

With great regularity a number of this learned and valuable work appears every two weeks. We have now before us parts 5, 6, and 7, completing the first volume, and furnishing 136 pages towards the second. The number of topics discussed in these three numbers is fifty, in which is included croup and feigned diseases, and all the maladies beginning with the letters between these two. In these articles will be found a summary of all the practical knowledge extant in relation to the diseases treated of. If some complain that it is not as full as it might have been made, we suspect that its compression within such reasonable limits will be rather a recommendation of the work with the physician in active practice. As a cyclopædia of practical medicine, when completed, it will constitute an invaluable repository of medical knowledge. We hope the publishers are rewarded for their enterprising spirit in bringing out the work in so creditable a style. Y.

